# The serum small non‐coding RNA (SncRNA) landscape as a molecular biomarker of age associated muscle dysregulation and insulin resistance in older adults

**DOI:** 10.1096/fj.202301089RR

**Published:** 2024-01-31

**Authors:** Mark A. Burton, Elie Antoun, Emma S. Garratt, Leo Westbury, Elaine M. Dennison, Nicholas C. Harvey, Cyrus Cooper, Harnish P. Patel, Keith M. Godfrey, Karen A. Lillycrop

**Affiliations:** ^1^ Human Development and Health Academic Unit, Faculty of Medicine University of Southampton Southampton UK; ^2^ NIHR Southampton Biomedical Research Centre University of Southampton and University Hospital Southampton NHS Foundation Trust Southampton UK; ^3^ MRC Lifecourse Epidemiology Centre University of Southampton Southampton UK; ^4^ Victoria University of Wellington Wellington New Zealand; ^5^ Academic Geriatric Medicine, Faculty of Medicine University of Southampton Southampton UK; ^6^ Biological Sciences University of Southampton Southampton UK

**Keywords:** ageing, epigenetics, HOMA2‐IR, insulin resistance, noncoding RNA, sarcopenia, serum

## Abstract

Small noncoding RNAs (sncRNAs) are implicated in age‐associated pathologies, including sarcopenia and insulin resistance (IR). As potential circulating biomarkers, most studies have focussed on microRNAs (miRNAs), one class of sncRNA. This study characterized the wider circulating sncRNA transcriptome of older individuals and associations with sarcopenia and IR. sncRNA expression including miRNAs, transfer RNAs (tRNAs), tRNA‐associated fragments (tRFs), and piwi‐interacting RNAs (piRNAs) was measured in serum from 21 healthy and 21 sarcopenic Hertfordshire Sarcopenia Study extension women matched for age (mean 78.9 years) and HOMA2‐IR. Associations with age, sarcopenia and HOMA2‐IR were examined and predicted gene targets and biological pathways characterized. Of the total sncRNA among healthy controls, piRNAs were most abundant (85.3%), followed by tRNAs (4.1%), miRNAs (2.7%), and tRFs (0.5%). Age was associated (FDR < 0.05) with 2 miRNAs, 58 tRNAs, and 14 tRFs, with chromatin organization, WNT signaling, and response to stress enriched among gene targets. Sarcopenia was nominally associated (*p* < .05) with 12 tRNAs, 3 tRFs, and 6 piRNAs, with target genes linked to cell proliferation and differentiation such as Notch Receptor 1 (*NOTCH1*), DISC1 scaffold protein (*DISC1*), and GLI family zinc finger‐2 (*GLI2*). HOMA2‐IR was nominally associated (p<0.05) with 6 miRNAs, 9 tRNAs, 1 tRF, and 19 piRNAs, linked with lysine degradation, circadian rhythm, and fatty acid biosynthesis pathways. These findings identify changes in circulating sncRNA expression in human serum associated with chronological age, sarcopenia, and IR. These may have clinical utility as circulating biomarkers of ageing and age‐associated pathologies and provide novel targets for therapeutic intervention.

## Graphical abstract

Small noncoding RNAs (sncRNAs), implicated in age‐associated pathologies, may act as important molecular biomarkers. Along with miRNAs, other sncRNA populations may provide novel insights into essential regulatory mechanisms of age‐associated disease. Differential expression of miRNA, tRNAs, and tRFs in older adult women were linked with genes within pathways involved in ageing. Furthermore, sncRNA populations associated with sarcopenia and HOMA2‐IR were linked with important regulatory networks suggesting sncRNAs in serum may have clinical utility as biomarkers of ageing and age‐associated pathologies.

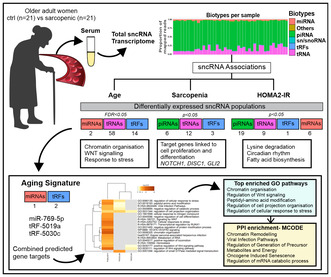

**Abbreviations**
ACTR3C: actin related protein 3C
ALMi: appendicular lean mass index
ATXN3: ataxin 3
BIRC5: baculoviral IAP repeat containing 5
C17orf103: N‐acetyltransferase domain containing 1
C2orf68: chromosome 2 open reading frame 68
CARD8: caspase recruitment domain family member 8
CCND1: cyclin D1
CDC6: cell division control protein 6
CFLAR: CASP8 and FADD like apoptosis regulator
CTGF: connective tissue growth factor
DISC1: disrupted in schizophrenia 1
FOXN3: forkhead Box N3
GLI2: GLI family zinc finger 2
Glut4: glucose transporter type 4
GO: gene ontology
HIF‐1a: hypoxia‐inducible factor 1α
HOMA2‐IR: homeostatic model assessment for insulin resistance
HSSe: hertfordshire sarcopenia study extension
IGF/PI3K/Akt: insulin growth factor/phosphoinositide 3‐kinase/Akt serine‐threonine protein kinase
IGF‐1: insulin‐like growth factor 1
IR: insulin resistance
KEGG: kyoto encyclopedia of genes and genomes
lncRNAs: long non‐coding RNAs
LYRM4: LYR motif containing 4
miRNAs: microRNAs
MYF5: myogenic differentiation 1
MYOD: myogenic differentiation 1
NOTCH1: notch receptor 1
PIAS2: protein inhibitor of activated STAT 2
piRNAdb: piRNA data base
piRNAs: piwi‐interacting RNAs
PTK7: protein tyrosine kinase 7
SIRT1: sirtuin‐1
sncRNAs: small noncoding RNAs
T2D: type 2 diabetes
TM2D3: TM2 domain containing 3
TMEM19: transmembrane protein 19
tRFdb: tRF data base
tRFs: tRNA‐associated fragments
tRNAs: transfer RNAs
VAT1L: vesicle amine transport 1 like

## 1 INTRODUCTION

Ageing is characterized by a gradual loss of general function, and reduced repair capacity which leads to an increased risk of mortality and susceptibility to multiple age‐related pathologies. Muscle ageing is associated with a progressive impairment in metabolic function and a loss of muscle mass. As skeletal muscle is critical for posture and is the primary organ implicated in glucose clearance, age related changes in muscle have been linked to both sarcopenia and insulin resistance. However, the underlying molecular mechanisms leading to sarcopenia or insulin resistance remain unclear.

Small non‐coding RNAs (sncRNAs) have been increasingly recognized as important regulators of many biological processes. sncRNA populations include microRNAs (miRNA), piwi‐interacting RNAs (piRNAs), transfer RNAs (tRNA), and tRNA‐associated fragments (tRFs). To date, the majority of research has focused on miRNAs which either repress translation or induce mRNA degradation of target transcripts through sequence‐specific binding to the 3′UTR. In skeletal muscle, miRNAs play key roles in muscle homeostasis, controlling muscle mass, function, and metabolism. For example, *miR‐675‐3p* and *miR‐675‐5p* promote muscle differentiation and regeneration by repressing the bone morphogenetic protein (BMP) pathway through targeting the anti‐differentiation SMAD transcription factors, SMAD1 and SMAD5 and the cell division control protein 6 (CDC6),^1^ *miR‐1* modulates muscle cell growth by regulating IGF/PI3K/Akt signaling by directly targeting *IGF‐1* ^2^ whilst *miR‐223* regulates glucose uptake by inhibiting *Glut4* in muscle tissue.^3^ miRNAs have also been implicated in muscle ageing through regulation of key genes including insulin‐like growth factors, FOXO transcription factors, myostatin, NAD‐dependent protein deacetylase sirtuin‐1 (SIRT1), and transforming growth factor‐β signaling pathways.^4^

miRNAs can be actively secreted from cells, either bound to RNA binding proteins,^5^ high‐density lipoproteins,^6^ or released during cell death,^7^ regulating mRNA targets in recipient cells and mediating cross talk between organs. The detection of circulating miRNAs has led to considerable interest in the use of miRNAs as circulating biomarkers of age‐related pathologies as well as targets for novel intervention strategies. For example, the first observation of altered circulating miRNA levels during aging was *miR‐34a*, which was elevated in the plasma of older mice.^8^ *miR‐34a* was also increased in peripheral blood mononuclear cells (PBMCs) and brains of older mice, with a reciprocal decrease of its target *Sirt1* mRNA. Furthermore, recent human studies have reported differential miRNA expression between young and aged individuals in several different peripheral fluids, including serum,^9^, ^10^, ^11^, ^12^ plasma,^13^, ^14^ and saliva.^15^ A number of studies have also reported differences in circulating miRNAs with respect to age associated pathologies including sarcopenia and insulin resistance. For example, eight miRNAs in plasma (*miR‐10a‐3p,−92a‐3p,−185‐3p,−194‐3p,−326,−532‐5p,−576‐5p*, and *−760*),^16^ and two miRNAs in serum (*miR‐21* and −*203a‐3p*)^17^ have been shown to be associated with sarcopenia status, while *miR‐29a, miR‐34a, miR‐375, miR‐103, miR‐107, miR‐132, miR‐142–3p*, and *miR‐144* have been identified as potential circulating biomarkers of type 2 diabetes.^18^

However, most studies investigating associations between circulating miRNAs and age‐associated pathologies have utilized targeted approaches to investigate altered miRNA expression, and the presence of sncRNA populations other than miRNAs in serum, and their potential as putative biomarkers has received little research attention. piRNAs function to maintain genome stability; in addition to their role in translation, tRNAs can regulate gene expression and have been shown to act as sensors of nutritional stress,^19^, ^20^, ^21^ while tRFs, generated through enzymatic cleavage of the 5′ or 3′ ends of tRNAs, demonstrate miRNA like activity, and have been reported to regulate gene expression by binding to the promoters of target genes.^22^, ^23^ Given the importance of these different classes of sncRNAs, we carried out global small RNA sequencing from serum samples from community dwelling older individuals with or without sarcopenia, to firstly characterize the sncRNA landscape of serum from aged individuals and subsequently investigate the associations of sncRNA expression levels with sarcopenia and insulin resistance; to date there have been limited studies investigating the association between circulating sncRNAs and these phenotypes. In addition, where possible we identified downstream gene targets of the sncRNAs, and pathways enriched amongst the target genes to gain insights into the underlying regulatory mechanisms altered during ageing and associated pathologies.

## 2 METHODS

### 2.1 Study participants

Participants were recruited from the UK Hertfordshire Sarcopenia Study extension (HSSe), designed to investigate life‐course influences on muscle function in community‐dwelling older people.^24^, ^25^, ^26^, ^27^ This study received ethical approval from the Hertfordshire Research Ethics Committee (number 07/Q0204/68) and was conducted in accordance with the 1964 Declaration of Helsinki and its later amendments. Sarcopenia status was defined according to the European Working Group on Sarcopenia in Older People (EWGSOP) 2010 definition,^28^ with the following thresholds: ALMi (ALM/height^2^) ≤ 7.23 kg/m^2^ for men and ≤5.67 kg/m^2^ for women; grip strength < 30 kg for men and <20 kg for women; and walking speed ≤ 0.8 m/s.

### 2.2 Serum sample processing and RNA extraction

To examine the association between circulating sncRNAs and ageing associated pathologies with a particular focus on muscle dysregulation, serum samples (*n* = 42) were selected from the HSSe cohort; this included serum samples from 21 women with sarcopenia, and from 21 women without sarcopenia, selected to be matched for age and HOMA2‐IR levels (Table 1). As the number of men with sarcopenia and sufficient serum for RNA extraction was limited, only serum samples from HSSe women participants were assessed in this study. Total RNA was extracted from serum samples using the miRNeasy Kit (Qiagen, UK) following manufacturers guidelines. RNA concentration and quality were quantified using Nanodrop and Qubit assays (Thermo Fisher, UK). Extracted RNA was treated with DNAse1 (Sigma, UK), snap frozen on dry ice, and stored at −80° until sequencing analysis.

**TABLE 1:** Participant characteristics.

Characteristics	Control (*n* = 21)	Sarcopenic (*n* = 21)	Total (*n* = 42)	*p*‐value
Age (years)	78.57 ± 2.49	79.15 ± 2.77	78.86 ± 2.62	.47
Height (cm)	160.65 ± 5.56	159.08 ± 5.20	158.87 ± 5.38	.35
Weight (kg)	73.01 ± 9.73	60.91 ± 9.54	66.97 ± 11.32	.0002***
BMI (kg/m^2^)	28.30 ± 3.49	24.02 ± 3.16	26.16 ± 3.94	<.0001****
HOMA2‐IR	0.88 ± 0.50	0.91 ± 0.49	0.90 ± 0.49	.82
Grip strength (kg)	22.19 ± 4.70	17.95 ± 4.84	20.07 ± 5.18	.0017**
Gait speed (m/s)	1.01 ± 0.19	0.88 ± 0.15	0.94 ± 0.18	.025*
AMLi (kg/m^2^)	6.10 ± 0.65	5.64 ± 0.40	5.64 ± 0.70	<.0001****

*Note*: Values are mean ± standard deviation.

Abbreviations: ALM, appendicular lean mass; ALMi, appendicular lean mass index; BMI, body mass index.

* *p* < .05;

** *p* < .01;

*** *p* < .001;

**** *p* < .0001.

### 2.3 sncRNA sequencing

Total RNA was used to perform small RNA‐seq (average approximately 18 million reads per sample) using the small RNA workflow (Oxford Genomics, OGC). RNA was quantified using RiboGreen (Invitrogen) on the FLUOstar OPTIMA plate reader (BMG Labtech) and the size profile and integrity analyzed on the 2200 or 4200 TapeStation (Agilent, RNA ScreenTape). Small RNA library preparation was completed using NEBNext Small RNA kit (NEB) following manufacturer's instructions and applying the low input protocol modifications. Libraries were amplified (15 cycles) on a Tetrad (Bio‐Rad) using in‐house unique dual indexing primers (based on DOI: 10.1186/1472‐6750‐13‐104). Size selection was performed using Pippin Prep instrument (Sage Science) using the 3% Agarose, dye free gel with internal standards (size selection: 125 to 160 bp). Individual libraries were normalized using Qubit, and size profile analyzed on the 2200 or 4200 TapeStation. The pooled library was diluted (~10 nM) for storage. The 10 nM library was denatured and further diluted prior to loading on the sequencer. Single end sequencing was performed using NextSeq500 platform (Illumina, NextSeq 500/550 v2.5 Kits, 75 cycles). BCL files were demultiplexed using bcl2fastq and fastq files generated. Adapters were trimmed using Trim Galore^29^ discarding reads that were <10 bp after trimming and any reads longer than 50 bp. For miRNA analysis, the miRDeep2^30^ pipeline was run. Briefly, reads were collapsed and aligned to the hg19 genome using the mapper.pl script, after which the miRDeep2.pl script was run to count both mature and hairpin miRNA species. Reads were aligned to tRFs using MINTmap.^31^ For all other sncRNA subtypes, fastq files were aligned to the hg19 genome using the short‐read aligner Bowtie^32^ using the following options: ‐q ‐k 10 ‐v 0 ‐S ‐t ‐‐best ‐‐strata. Gene counts were generated using featureCounts.^33^

### 2.4 Small RNAseq data processing and analysis

Sequencing data was analyzed using voom and limma, applying voom transformation to the normalized and filtered dataset with between‐array normalization (quantile normalization) after which models were fitted with limma. Although the age range of the participants was narrow, an initial analysis was performed to determine whether age influenced the serum sncRNA profile. As this analysis showed significant changes in the sncRNA transcriptome with respect to chronological age, subsequent analyses investigating the sncRNA profile with regards to sarcopenia and HOMA2‐IR were adjusted for age. After data processing and removal of lowly expressed genes, 112 miRNAs, 359 tRNAs, 68 tRFs, and 382 piRNAs remained for further analysis. Outliers were determined visually from PCA plots. The sncRNAseq data can be accessed on the gene expression omnibus (https://www.ncbi.nlm.nih.gov/geo/), under accession number GSE231785.

### 2.5 sncRNA predicted targets and biological significance

#### 2.5.1 miRNAs target information and pathway enrichment

miRNA target genes and their related functional pathways were identified using DIANA‐mirPath v3.0 (http://snf‐515788.vm.okeanos.grnet.gr/).^34^ Predicted and/or experimentally validated gene targets were identified within mirPath software using Tarbase v7.0, the largest manually curated, experimentally validated miRNA–gene interactions database.^35^ Identified targets were subsequently used for KEGG and Gene Ontology pathway analysis.

#### 2.5.2 tRF target information and gene ontologies

To determine the functional role of tRFs, differentially expressed tRFs were entered into the tRF data base (tRFdb) (http://genome.bioch.virginia.edu/trfdb/) to identify known sequence information. tRForest (https://trforest.com/) was used to explore machine‐learning predicted tRF gene targets and identify tRF associated gene ontologies (GO). For the GO output a tRForest generated dotplot was created showing the ten pathways with the highest gene ratios and to display data for the gene ratio, gene count, and adjusted *p*‐value. Furthermore, a network plot was created using the cnetplot function, showing connections between genes and the highest‐ranking pathways. Novel tRFs identified in the expression data that were not currently present in the tRFdb were reported with no predicted experimentally validated gene target interactions or associated ontologies.

#### 2.5.3 Determining the targets and gene ontologies of differentially expressed piRNAs

piRNA targets were investigated using piRNAdb (https://www.pirnadb.org/about/informations/database).

#### 2.5.4 Combining different sncRNA population target genes to identify enriched pathways

miRNA and tRF target genes identified using mirPath or tRForest were combined and investigated using Metascape.^36^ Briefly, for each gene target list, pathway process enrichment was carried out. Terms with a *p*‐value <.01, a minimum of three genes, and an enrichment factor >1.5 were used.^37^ Protein–protein interaction (PPI) enrichment analysis was performed along with the molecular complex detection (MCODE) algorithm^38^ in Metascape to identify densely connected network components.

### 2.6 Statistical analysis

All statistical analyses were carried out in R (version 3.4.2) and GraphPad Prism (v10.0.3). Demographic characteristics were compared between controls and those with sarcopenia for normality using the Shapiro–Wilk test. Normally distributed data was compared using unpaired *t* test and non‐normally distributed using the Mann–Whitney test. mirPath enrichment analysis was performed using Fishers exact test (hypergeometric distribution). Metascape *p*‐values were calculated based on the cumulative hypergeometric distribution, and *q*‐values calculated using Benjamini‐Hochberg.

## 3 RESULTS

### 3.1 Participant characteristics

Participant characteristics are summarized in Table 1. RNA was extracted from serum samples from 42 women from the HSSe; this comprised 21 individuals diagnosed with sarcopenia, with a mean age of 79.15 (± SD 2.77); these were the only individuals with sarcopenia in the HSSe cohort who had sufficient serum available for RNA extraction. 21 individuals without sarcopenia were also selected from the HSSe cohort, these were matched with the sarcopenic participants for age (mean age of 78.57.1 (± SD 2.49)) and HOMA2‐IR levels (0.88 ± SD 0.50). Sarcopenic participants had a mean grip strength (kg) of 17.95 (± SD 4.84), gait speed (m/s) of 0.88 (±SD 0.15), and ALMi (kg/m^2^) of 5.64 (±SD 0.40) compared to controls with a mean grip strength (kg) of 22.19 (±SD 4.70), gait speed (m/s) of 1.01 (±SD 0.19), and ALMi (kg/m^2^) of 6.10 (±SD 0.65).

### 3.2 Baseline sncRNA abundance in human serum from older individuals

**FIGURE 1:**
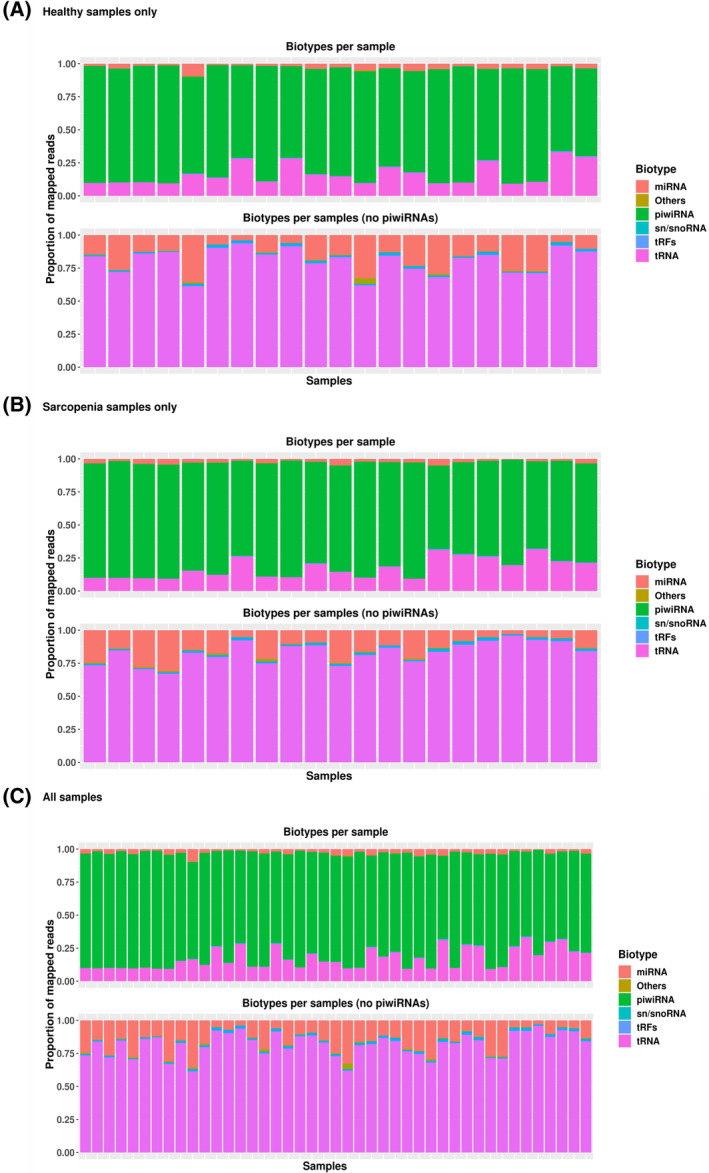
(A) Relative abundance (read counts) of sncRNA subtypes in serum from control participants (*n* = 21). piRNAs were the most abundant, followed by tRNA, miRNA, tRFs, and sn/snoRNA. Misc (“other”) RNA included additional small RNA subtypes not yet annotated in available sncRNA databases. (B) Relative abundance of sncRNA subtypes identified in sarcopenics (*n* = 21) with piRNAs most abundant (84.8%), followed by tRNAs (4.04%), miRNAs (2.81%), and tRFs (0.57%). (C) Relative abundance of sncRNA subtypes identified in controls and sarcopenics (*n* = 42).

sncRNA profiles of control serum samples (*n* = 21) were used to determine the abundance of the different classes of sncRNA based on absolute read counts. piRNAs were the most abundant (85.3%) sncRNA species detected in serum, followed by tRNAs (4.06%), miRNAs (2.74%), and tRFs (0.54%) (Figure 1A). Within the sarcopenic participants (*n* = 21) the overall abundances of sncRNAs were similar to controls, with piRNAs the most abundant (84.8%), followed by tRNAs (4.04%), miRNAs (2.81%), and tRFs (0.57%) (Figure 1B,C).

### 3.3 Differentially expressed miRNAs, tRNAs, and tRFs were significantly associated with chronological age

**FIGURE 2:**
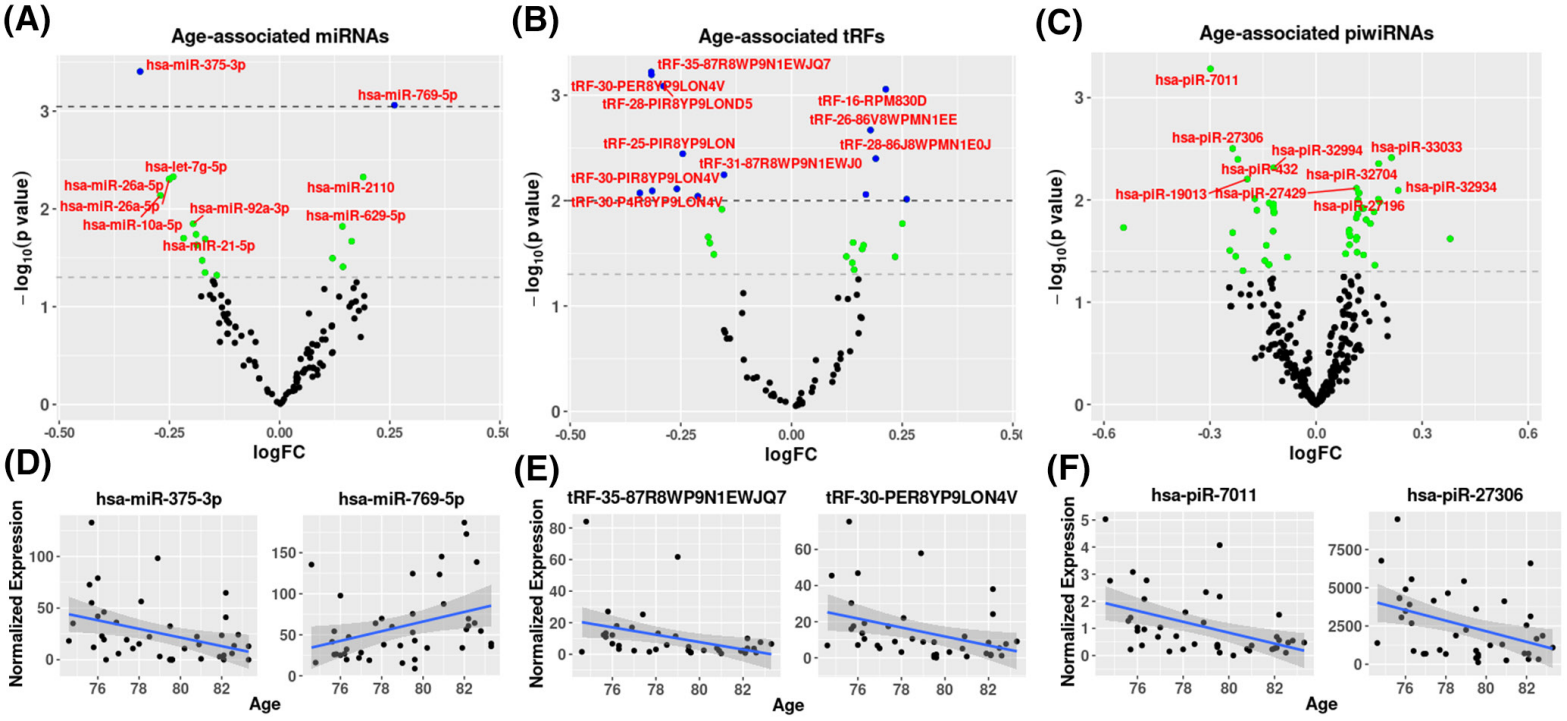
Volcano plots of age associated (FDR < 0.05) miRNAs (A), tRFs, (B) and (*p* < .05) piRNAs (C). Regression graphs of top two differentially expressed miRNAs (D), tRFs (E), piRNAs (F), respectively. For volcano plots differentially expressed FDR (FDR < 0.05) associated sncRNAs are in blue, nominally associated (*p* < .05) sncRNAs are in green and all others are in black. Dashed black line represents an FDR < 0.05 and the dashed grey line represents *p*‐value < .05.

The association between sncRNA expression and chronological age was initially examined across all participants, as although there was a narrow age range of participants in this study, previous studies have shown that age is a significant driver of changes in circulating miRNA levels. Significant associations between serum sncRNA expression and age were identified. There were 2 miRNAs associated (FDR < 0.05) with age (Figure 2; Tables 2 and S1), *miR‐375/miR‐375‐3p* (FDR = 4.4 × 10^−2^) whose expression decreased with age, and *miR‐769/miR769‐5p* (FDR = 4.8 × 10^−2^) whose expression increased with age. There were significant associations between tRNA and tRF expression and age, with 58 tRNAs (FDR < 0.05) and 14 tRFs (FDR < 0.05), differentially expressed (Figures 2 and S1; Tables 2 and S2,S3). The top three differentially expressed tRNAs were *tRNA‐Gly‐CCC‐4‐1* (FDR = 8.9 × 10^−3^), *tRNA‐Lys‐CTT‐2‐3* (FDR = 8.9 × 10^−3^), and *tRNA‐Lys‐CTT‐2‐1* (FDR = 8.9 × 10^−3^), with the top three tRFs identified as *tRF‐35‐87R8WP9N1EWJQ7*, *tRF‐30‐PER8YP9LON4V*, and *tRF‐28‐PIR8YP9LOND5*. No piRNAs passed FDR significance (FDR < 0.05), however, 57 piRNAs were nominally associated (*p* < .05) with age (Tables 2 and S4; Figure 2).

**TABLE 2:** Differentially expressed (FDR < 0.05) or (*p* < .05) miRNA's, tRNA's, tRF's, and piRNA's (top three) associated with age, sarcopenia, or IR. Signfiance values with a p or FDR value ≤.05 are shown in bold.

sncRNA	logFC	*p*‐value	FDR
*Age associated sncRNAs*			
miRNA			
miR‐375‐3p_mir‐375	−0.31640	3.93 × 10^−04^	**4.40 × 10** ^**−02**^
miR‐769‐5p_mir‐769	0.26066	8.70 × 10^−04^	**4.87 × 10** ^**−02**^
tRNA			
tRNA‐Gly‐CCC‐4‐1	−0.24853	4.10 × 10^−05^	**8.91 × 10** ^**−03**^
tRNA‐Lys‐CTT‐2‐3	−0.17392	1.43 × 10^−04^	**8.91 × 10** ^**−03**^
tRNA‐Lys‐CTT‐2‐1	−0.17085	1.74 × 10^−04^	**8.91 × 10** ^**−03**^
tRF's			
tRF‐35‐87R8WP9N1EWJQ7	−0.31674	6.01 × 10^−04^	**1.49 × 10** ^**−02**^
tRF‐30‐PER8YP9LON4V	−0.31622	6.39 × 10^−04^	**1.49 × 10** ^**−02**^
tRF‐28‐PIR8YP9LOND5	−0.28901	8.20 × 10^−04^	**1.49 × 10** ^**−02**^
piRNA's			
piR‐7011	−0.29885	**0.013644**	1.94 × 10^−01^
piR‐27 306	−0.23599	**0.017044**	1.94 × 10^−01^
piR‐33 033	0.21328	**0.024331**	1.94 × 10^−01^
*Sarcopenia associated sncRNAs*			
tRNA			
tRNA‐Cys‐GCA‐14–1	0.88444	**7.41 × 10** ^**−03**^	9.96 × 10^−01^
tRNA‐Phe‐GAA‐1‐1	−0.84558	**1.57 × 10** ^**−02**^	9.96 × 10^−01^
tRNA‐Thr‐TGT‐6‐1	−0.92106	**2.56 × 10** ^**−02**^	9.96 × 10^−01^
tRF's			
tRF‐16‐3JWB61B	−1.3598	**3.08 × 10** ^**−02**^	9.97 × 10^−01^
tRF‐28‐P4R8YP9LOND5	1.2988	**3.28 × 10** ^**−02**^	9.97 × 10^−01^
tRF‐31‐P4R8YP9LON4VD	1.1350	**4.54 × 10** ^**−02**^	9.97 × 10^−01^
PiRNA's			
piR‐29 608	−1.4573	**1.36 × 10** ^**−02**^	9.98 × 10^−01^
piR‐10 750	−0.7154	**1.70 × 10** ^**−02**^	9.98 × 10^−01^
piR‐2139	−1.0146	**2.43 × 10** ^**−02**^	9.98 × 10^−01^
*HOMA2‐IR associated sncRNAs*			
miRNA			
miR‐1908‐5p_mir‐1908	−2.5195	**4.37 × 10** ^**−03**^	4.90 × 10^−01^
miR‐146b‐5p_mir‐146b	2.2630	**2.32 × 10** ^**−02**^	6.13 × 10^−01^
miR‐4508_mir‐4508	−2.6053	**2.44 × 10** ^**−02**^	6.13 × 10^−01^
tRNA			
tRNA‐Val‐CAC‐3‐1	0.7115	**1.31 × 10** ^**−02**^	9.72 × 10^−01^
tRNA‐Lys‐CTT‐chr16‐1	−1.7758	**3.28 × 10** ^**−02**^	9.72 × 10^−01^
tRNA‐Lys‐TTT‐13–1	−1.3963	**3.44 × 10** ^**−02**^	9.72 × 10^−01^
tRF's			
tRF‐16‐Q1Q89PE	−2.17388	**3.81 × 10** ^**−02**^	7.70 × 10^−01^
piRNA's			
piR‐27 696	−1.7900	**1.64 × 10** ^**−02**^	6.27 × 10^−01^
piR‐18 752	−3.4780	**4.87 × 10** ^**−02**^	7.60 × 10^−01^
piR‐21 284	2.4275	**1.32 × 10** ^**−02**^	7.60 × 10^−01^

#### 3.3.1 Predicted gene target and pathway analysis of chronological age associated sncRNAs are associated with extracellular matrix (ECM) receptor interaction, components of energy regulation, and cancer pathways

To gain insights into the functional significance of the age associated sncRNAs, the target genes of each class of sncRNA was investigated. DIANA miRPath was used to identify validated target gene interactions for the differentially expressed miRNAs associated with age and the pathways enriched amongst the target genes. *miR‐375/miR‐375‐3p* was not present in the DIANA miRPath database, however for *miR‐769/miR769‐5p*, 319 gene interactions were identified, and seven KEGG pathways enriched amongst the target genes (Table 3). The top three enriched pathways associated with *miR‐769/miR‐769‐5p* were ECM‐Receptor Interaction (*p* < 1 × 10^−325^), lysine degradation (*p* = 3.09 × 10^−6^), and pantothenate and CoA biosynthesis (*p* = 2.0 × 10^−3^) comprising 6, 5, and 2 target genes, respectively.

**TABLE 3:** DIANA mirPath v.3 pathway analysis (genes union) of differentially expressed miRNAs associated with age (FDR < 0.05) or IR (*p* < .05) from serum of older individuals.

KEGG pathway	*p*‐value	genes	miRNAs
*Age associated miRNA (available in DIANA miRPath)*			
miR 769‐5p			
ECM‐receptor interaction	3.94 × 10^−21^	6	1
Lysine degradation	3.09 × 10^−06^	5	1
Pantothenate and CoA biosynthesis	2.09 × 10^−03^	2	1
Sulfur metabolism	2.79 × 10^−02^	1	1
Amoebiasis	2.85 × 10^−02^	6	1
Glioma	2.85 × 10^−02^	5	1
Central carbon metabolism in cancer	2.85 × 10^−02^	4	1
*IR associated miRNAs (top 2 with associated pathways)*			
miR‐146b‐5p			
Lysine degradation	1.76 × 10^−02^	4	1
Circadian rhythm	1.76 × 10^−02^	5	1
2‐Oxocarboxylic acid metabolism	2.24 × 10^−02^	2	1
miR‐4508			
Ribosome	3.96 × 10^−03^	1	1

**FIGURE 3:**
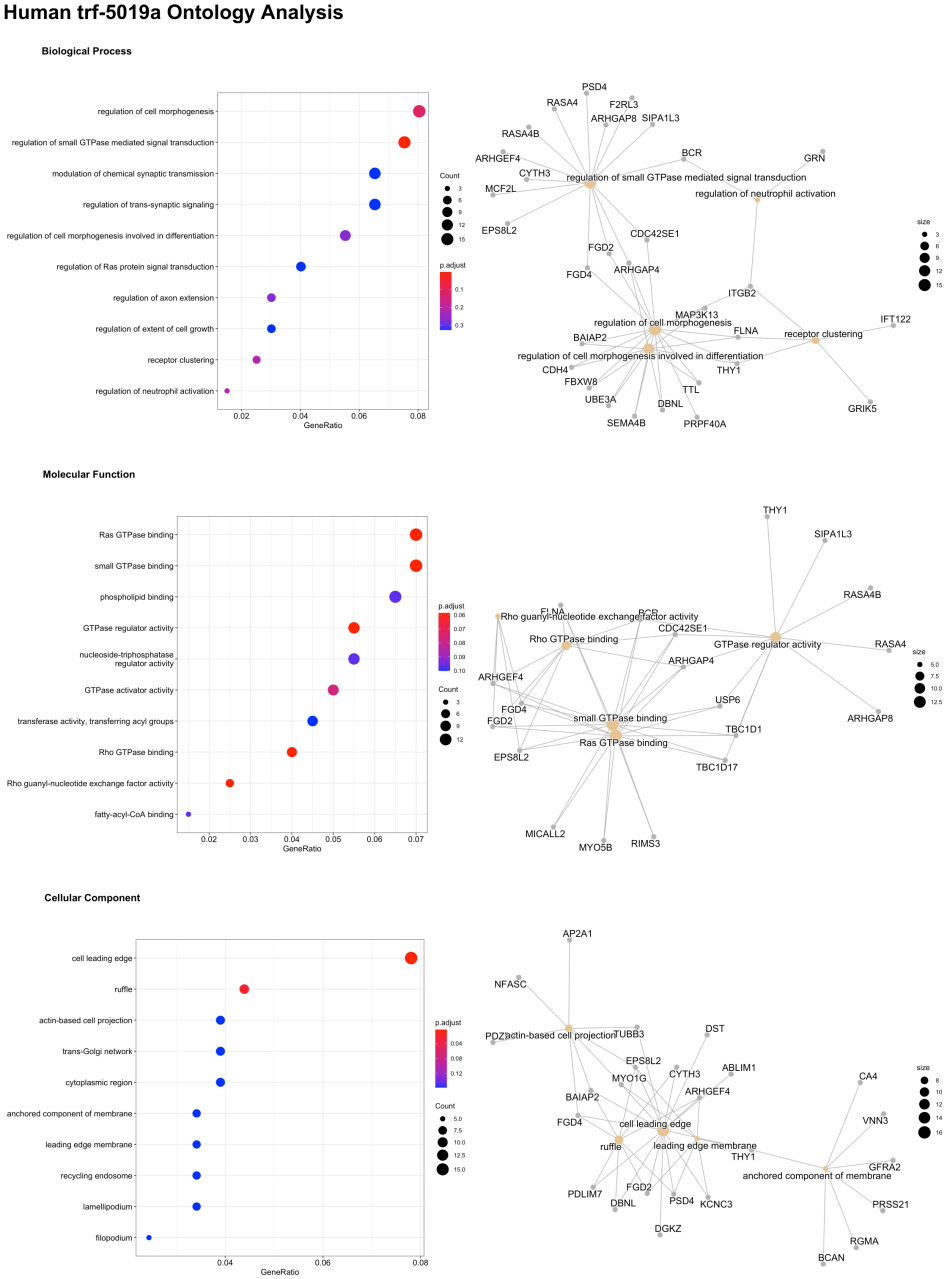
trForest Gene Ontology analysis of tRF‐16‐RPM830D (trf‐5019a) significantly (FDR < 0.05) associated with age in serum from older individuals.

Of the 14 differentially expressed (FDR < 0.05) tRFs associated with chronological age, 2 were identified by sequence homology in the tRF database, these being *tRF‐16‐RPM830D* (*tRF‐5019a*) (FDR = 1.4 × 10^−2^), and *tRF‐31‐87R8WP9N1EWJ0* (*trf‐5030c*) (FDR = 4.7 × 10^−2^) with the remaining 12 currently uncharacterized in the database. tRForest identified 248 unique transcript targets and 170 unique gene targets for *tRF‐5019a* (Table S5). The top three gene targets of *tRF‐5019a* were identified as transmembrane protein 19 (*TMEM19*, prediction score 1), TM2 Domain Containing 3 (*TM2D3*, prediction score 0.995), and Forkhead Box N3, (*FOXN3*, prediction score 0.99). Gene ontology analysis of the pathways significantly enriched for target genes of *tRF‐5019a* in the molecular function (MF) category were Ras GTPase binding (Figure 3). Analysis of *tRF‐5030c* identified 185 unique transcript targets and 97 unique gene targets (Table S6). The top three gene targets were identified as LYR motif containing 4 (*LYRM4*, prediction score = 1), N‐acetyltransferase domain containing 1 (c17orf103, prediction score = 1) and protein tyrosine kinase 7 (*PTK7*, prediction score = 1). There were no significantly enriched pathways amongst *tRF‐5030c* target genes (Table S7).

Using the piRNA data base, piRNAdb, the analysis of the most significant (*p* < .05) piRNA associated with chronological age (*piR‐7011*) identified 191 alignments within the human genome (hg38). Top predicted gene targets of *piR‐7011* based on the number of complementary sites to the piRNA sequence were identified as Ataxin 3 (*ATXN3*, 31 target sites), caspase recruitment domain family member 8 (*CARD8*, 17 target sites), actin related protein 3C (*ACTR3C*, 14 target sites), CASP8 and FADD like apoptosis regulator (*CFLAR*, 13 target sites), and protein inhibitor of activated STAT 2 (*PIAS2*, 16 target sites).

### 3.4 Differentially expressed tRNAs, tRFs, and piRNAs were nominally associated with sarcopenia status

**FIGURE 4:**
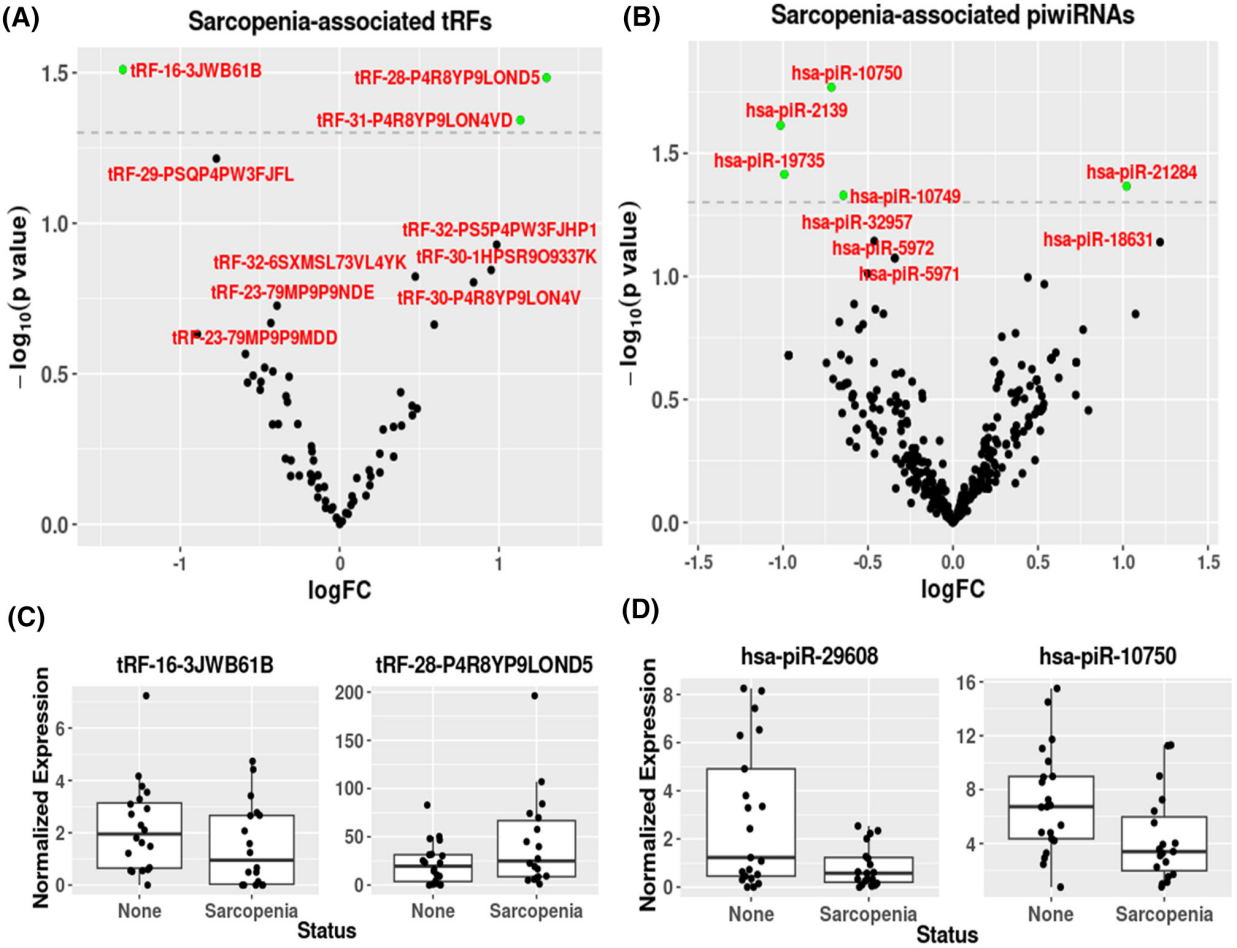
Volcano plots of sarcopenia associated (*p* < .05) (A) tRFs and (B) piRNAs. Regression graphs of top two differentially expressed (C) tRNAs and (D) piRNAs, respectively. For volcano plots, differentially expressed (*p* < .05) sncRNAs are in green and all others in black. Dashed line represents *p* = .05.

To identify potential sncRNA biomarkers of sarcopenia, the relationship between circulating sncRNAs and sarcopenia status was assessed, after adjustment for age (Figure 4). Sarcopenia was associated with 12 tRNAs and 3 tRFs at a *p* value <.05 but these associations did not remain after adjusting for multiple testing (Figures 4 and S1; Tables S8 and S9). The top three differentially expressed tRNAs with respect to sarcopenia status were *tRNA‐Cys‐GCA‐14–1* (*p* = 7.4 × 10^−3^), *tRNA‐Phe‐GAA‐1‐1* (*p* = 1.5 × 10^−2^), and *tRNA‐Thr‐TGT‐6‐1* (*p* = 2.5 × 10^−2^), while the three differentially expressed tRFs were *tRF‐16‐3JWB61B* (*p* = 3.1 × 10^−2^) (FC = 1.845), *tRF‐28‐P4R8YP9LOND5* (*p* = 3.3 × 10^−2^), and *tRF‐31‐P4R8YP9LON4VD* (*p* = 4.5 × 10^−2^) (Figure 4). Six piRNAs were associated with sarcopenia status (*p* ≤ .05), with the top three identified as *piR‐29 608* (*p* = 1.4 × 10^−2^), *piR‐10 750* (*p* = 1.7 × 10^−2^), and *piR‐2139* (Figure 4 and Table S10). There were no miRNAs associated with sarcopenia with a *p* value of <.05 (Table S11).

#### 3.4.1 Predicted gene target and pathway analysis of sarcopenia associated sncRNAs are associated with oxidoreductase activity and neural precursor cell proliferation/regulation

**FIGURE 5:**
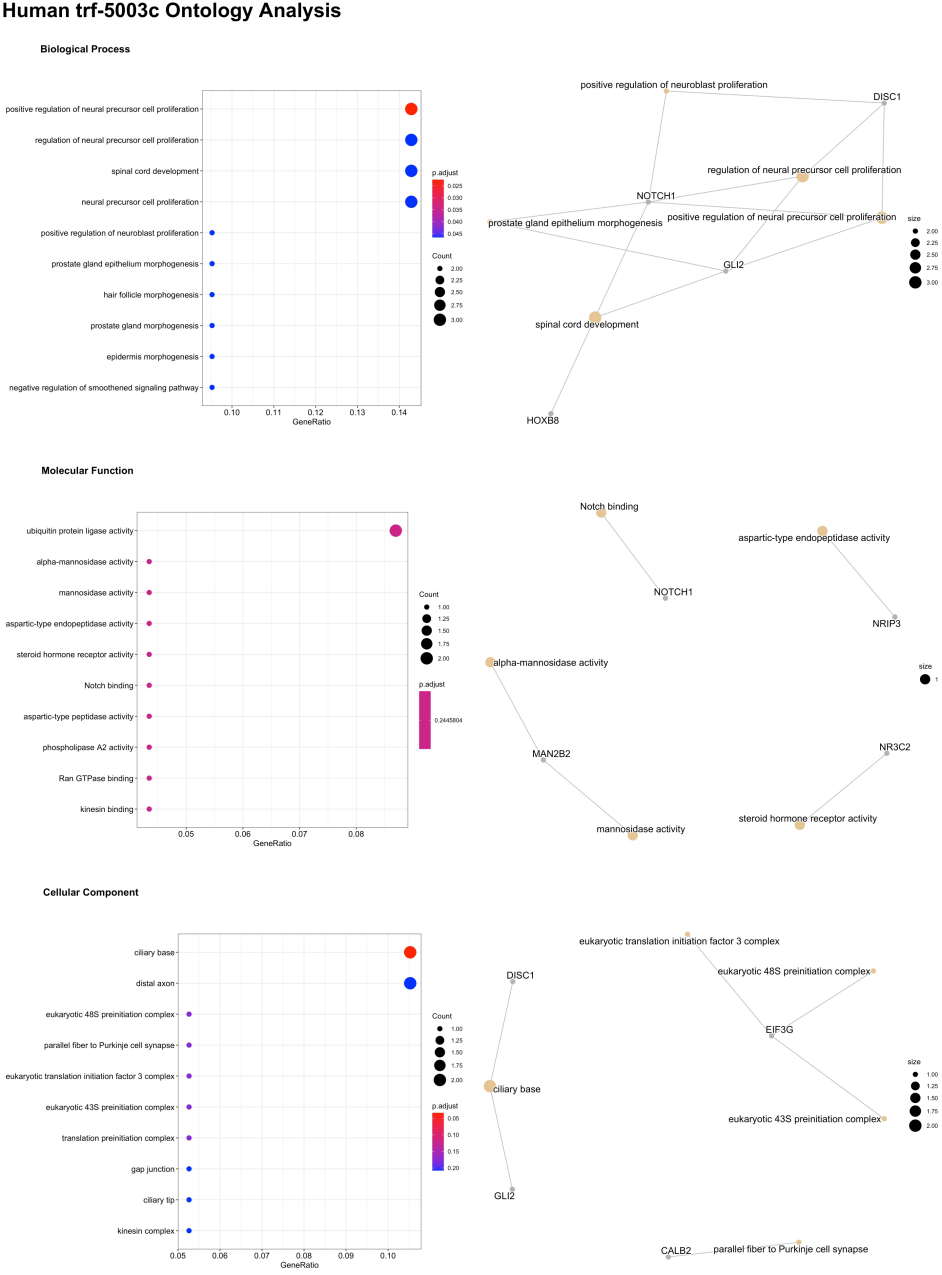
trForest Gene Ontology analysis of tRF‐31‐P4R8YP9LON4VD (trf‐5003c) significantly (*p* < .05) associated with sarcopenia in serum from older individuals.

Of the three nominally significant (*p* < .05) tRFs associated with sarcopenia, *tRF‐31‐P4R8YP9LON4VD* was the only tRF with sequence homology identified in the tRFdb (*tRF‐5003c*). Analysis of tRF‐5003c identified 32 unique gene transcripts and 24 unique genes of which the top three gene targets were chromosome 2 open reading frame 68 (*C2orf68*) (prediction score = 1), vesicle amine transport 1 like *(VAT1L)* (prediction score = 0.99) and disrupted in schizophrenia 1 (*DISC1*) (prediction score = 0.985) (Table S12). There was significant enrichment of pathways in the biological process category for *tRF‐5003c*, with pathways involved in positive regulation of neural precursor cell proliferation, regulation of neural precursor cell proliferation and spinal cord development being enriched, primarily driven by *NOTCH1*, *DISC1*, and *GLI2* being targets of *trf‐5003c*, which play key roles in cell proliferation and differentiation including myogenesis (Figure 5 and Table S13).

### 3.5 Differentially expressed miRNAs, tRNAs, tRFs, and piRNAs were nominally associated with HOMA2‐IR

**FIGURE 6:**
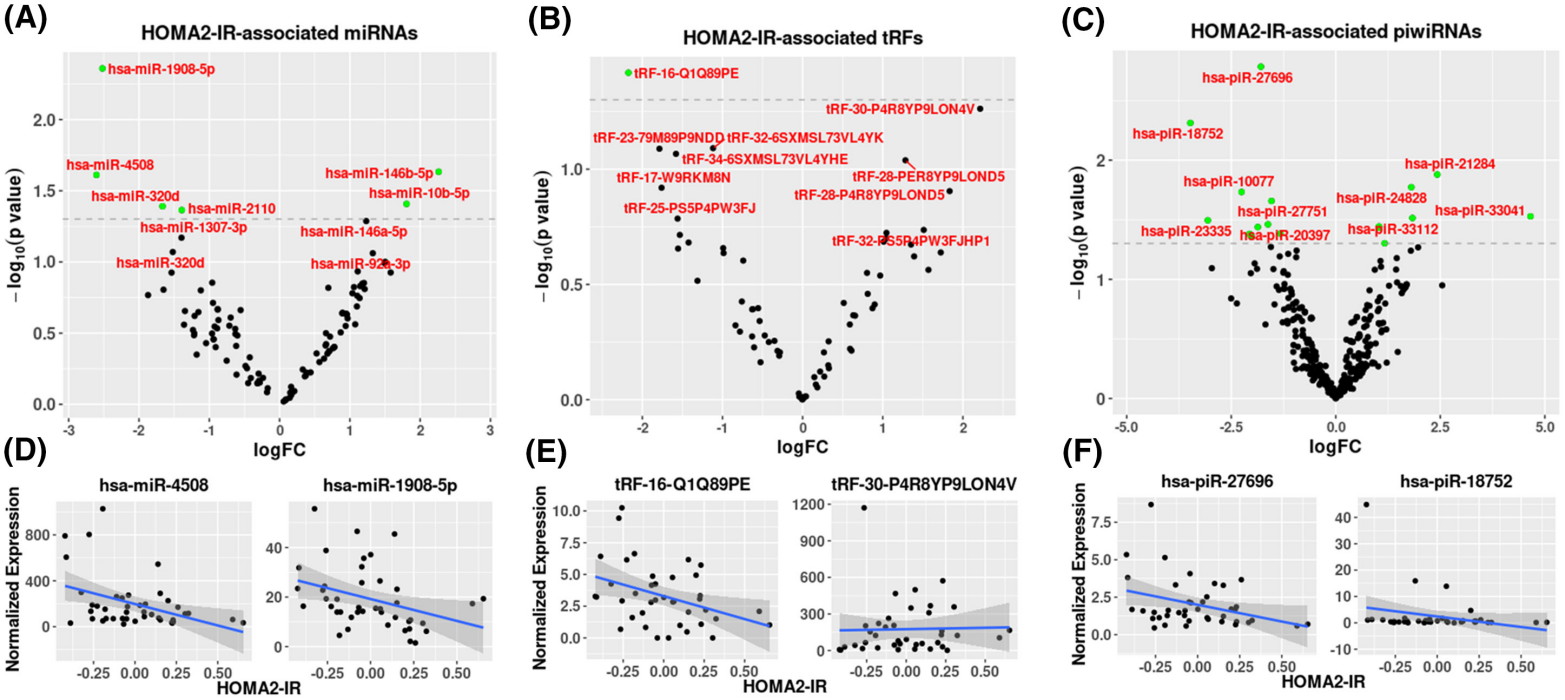
Volcano plots of HOMA2‐IR associated (*p* < .05) miRNAs (A), tRFs, (B), and piRNAs (C). Top two differentially expressed, miRNAs (D), tRFs (E), piRNAs (F), respectively. For volcano plots differentially expressed (*p* < .05) sncRNAs are in green and all others in black. Dashed grey line represents *p* = .05.

HOMA2‐IR was nominally (*p* < .05) associated with six miRNAs of which the top three were *miR‐1908‐5p/mir‐1908* (*p* = 4.3 × 10^−3^) *miR‐4508/miR‐4508* (*p* = 2.3 × 10^−2^), and *miR‐146b‐5p/mir‐146b* (*p* = 2.4 × 10^−2^) (Figure 6 and Table S14). Furthermore, HOMA2‐IR was nominally associated with nine tRNAs, of which the top three were *tRNA‐Val‐CAC‐3‐1* (*p* = 1.3 × 10^−2^) (FC 1.63), *tRNA‐Lys‐CTT‐chr16‐1* (*p* = 23.2 × 10^−2^) (FC = 0.292), and *tRNA‐Lys‐TTT‐13‐1* (*p* = 3.4 × 10^−2^); as well as 1 tRF identified as *tRF‐16‐Q1Q89PE* (*p* = 3.8 × 10^−2^) (Figures 6 and S1; Tables S15 and S16). 19 piRNAs were also nominally associated with HOMA2‐IR of which the top three were identified as *piR‐27 696* (*p* = 1.64 × 10^−3^), *piR‐18 752* (*p* = 4.8 × 10^−3^), and *piR‐21 284* (*p* = 1.3 × 10^−2^) (FC = 5.379) (Figure 6 and Table S17).

#### 3.5.1 Predicted gene target and pathway analysis of HOMA2‐IR associated sncRNAs are associated with lysine degradation, circadian rhythm, and fatty acid biosynthesis

**FIGURE 7:**
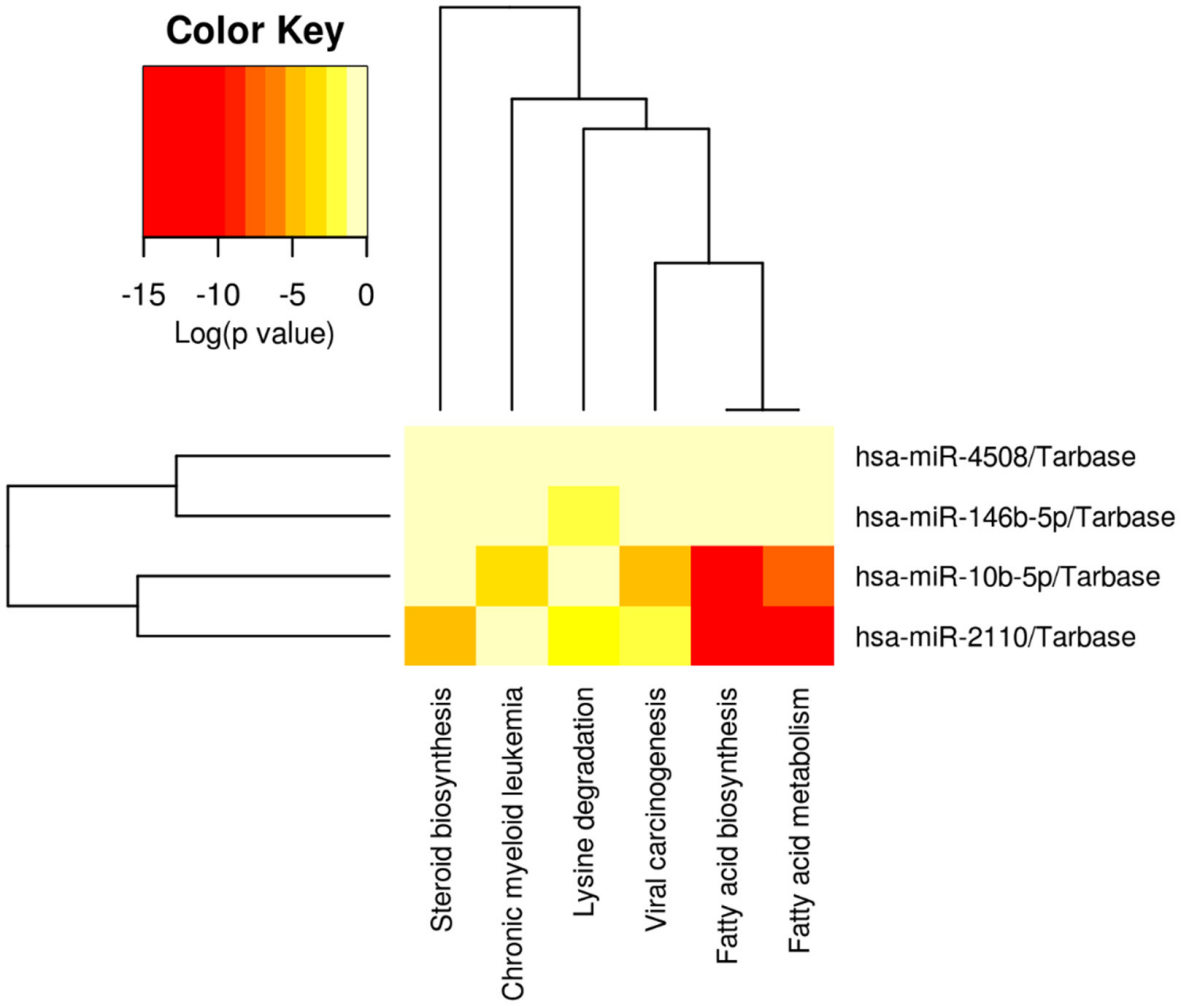
Diana mirPath v3 GO (KEGG) pathway union enrichment analysis of miRNAs nominally (*p* < .05) associated with HOMA2‐IR.

DIANA miRPath identified associated gene targets and pathways for nominally expressed miRNAs associated with HOMA2‐IR. There were no gene targets identified for *miR‐1908‐5p/miR‐1908*, however, for *miR‐146b‐5p/miR‐146b*, *p* = 2.3 × 10^−2^, 478 gene targets and 3 KEGG pathways were identified which included lysine degradation (*p* = 1.76 × 10^−2^), circadian rhythm (*p* = 1.76 × 10^−2^) and 2 oxocarboxilic acid metabolism (*p* = 2.24 × 10^−2^) (Table 3). Combining four of the six nominally significant miRNAs with identified gene targets in DIANA mirPath showed significant enrichment within six pathways (pathway union) (Figure 7 and Table S18). The top three enriched pathways were identified as fatty acid biosynthesis (*p* < 1 × 10^−325^), fatty acid metabolism (*p* = 5.44 × 10^−15^), and viral carcinogenesis (*p* = 3.4 × 10^−4^) and the top pathway with the most miRNAs that overlap (2) was fatty acid biosynthesis (*p* < 1 × 10^−325^). The tRF‐*Q1Q89PE* associated with HOMA2‐IR levels was not characterized in the tRF data base and its targets at present are unknown.

#### 3.5.2 Combining sncRNA target genes to identify putative regulatory networks

**FIGURE 8:**
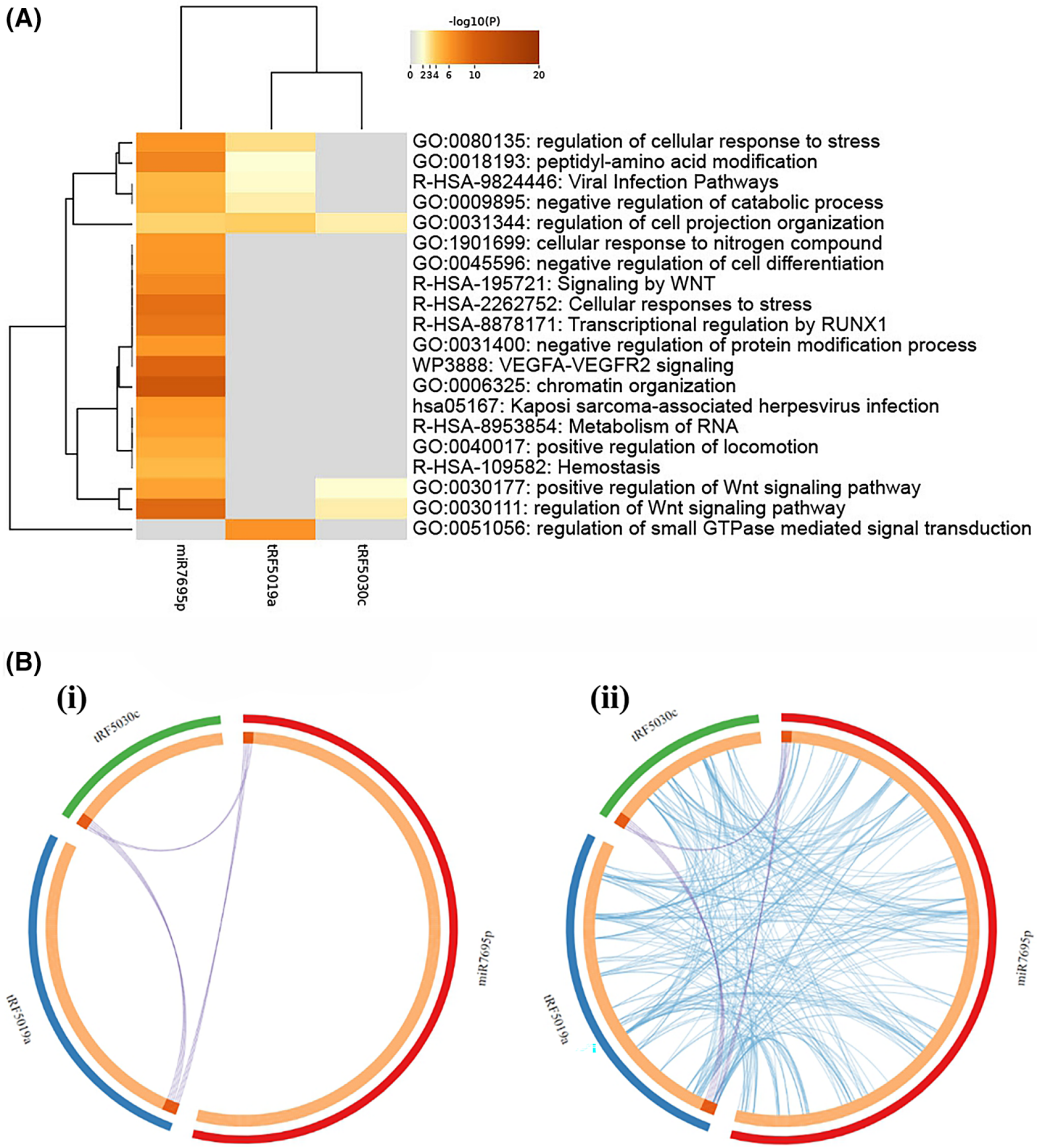
(A) Heat map of age associated sncRNA enriched pathways (top 20) across input predicted target gene lists colored by *p*‐values with darker orange representing greater significance. (B) Circos plots showing the overlaps between sncRNA target gene lists (i) at the gene level (purple links identical genes), (ii) including the shared term level, with linked genes (blue) belonging to the same enriched ontology term. The inner circle represents gene lists, with hits arranged along the arc. Genes that hit multiple lists are colored in dark orange, and genes unique to a list are shown in light orange.

**FIGURE 9:**
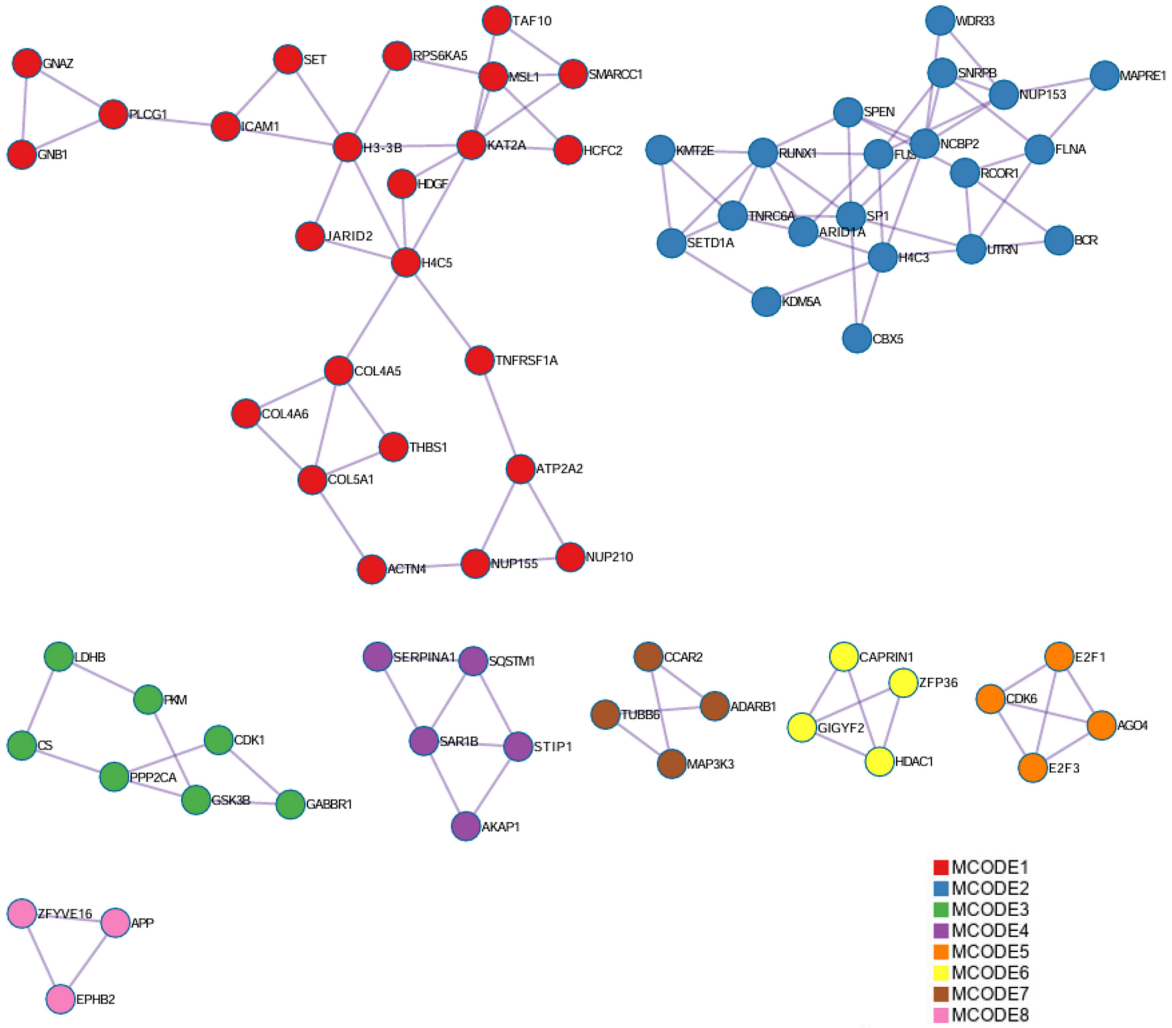
Molecular complex detection (MCODE) algorithm run against PPI enrichment networks from combined age associated sncRNAs predicted target genes to identify densely connected network components. The MCODE networks identified for combined gene lists (8) are shown colored by cluster.

To explore collective gene signatures encompassing the different sncRNA populations associated with age, sarcopenia, and HOMA2‐IR, we analysed the sncRNA predicted gene targets associated with each phenotype and performed pathway enrichment analysis. For age associated sncRNAs, the top differentially expressed miRNA present within miRpath (*miR769‐5p*) and the top tRFs present in trfDB (*tRF‐16‐RPM830D* (*tRF‐5019a*) and *tRF‐31‐87R8WP9N1EWJ0* (*tRF‐5030c*)) which had identifiable gene targets were combined and analyzed using Metascape^36^ (Table S19). Overlap was observed between the different sncRNAs target genes and enriched ontology pathways (Figure 8A,B). Top enriched GO pathways for this combined age associated sncRNA signature were identified as chromatin organization (Log10(*q*) −7.39), regulation of Wnt signaling pathway (Log10(*q*) −5.47), peptidyl‐amino acid modification (Log10(*q*) −4.81), regulation of cell projection organization (Log10(*q*) −4.77) and regulation of cellular response to stress (Log10(*q*) −3.96) (Table S20). Further analysis investigating protein–protein interaction (PPI) networks was also performed in Metascape, this identified chromatin organization (Log10(*p*) −15.5) and chromatin remodeling (Log10(*p*) −13.4) (GO Biological Process) along with Cellular Response to stress (Reactome Gene Set) (Log10(*p*) −15.2) as PPI networks amongst the gene targets of the age associated sncRNAs. To identify the nodes embedded in the large PPI networks MCODE analysis was conducted and putative biological roles of each MCODE complex assigned. MCODE analysis identified 8 densely connected network components (Figure 9). Pathway and process enrichment analysis of each MCODE component identified chromatin remodelling (MCODE_1, Log10(*p*) −12.3), viral infection pathways (MCODE_2, Log10(*p*) −6.4), regulation of generation of precursor metabolites and energy (MCODE_3, Log10(*p*) −5.5), oncogene induced senescence (MCODE_5, Log10(*p*) −11.9) and regulation of mRNA catabolic process (MCODE_6, Log10(*p*) −5.6) as the most significant pathways (Table S21). For Sarcopenia and HOMA2‐IR associated sncRNAs, either no miRNAs were differentially expressed (sarcopenia) or it was not possible to acquire gene targets from the database for both miRNAs and tRFs to perform a combined analysis.

## 4 DISCUSSION

Here, we report differential expression of sncRNAs in human serum from older community dwelling women with respect to chronological age, sarcopenia status and HOMA2‐IR. SncRNAs were strongly associated with age, with their gene targets enriched in chromatin organization, WNT signaling and response to stress. Furthermore, the sarcopenia associated sncRNAs included gene targets involved in cell proliferation and differentiation, while the HOMA2‐IR associated sncRNAs targeted lysine degradation, circadian rhythm, and fatty acid biosynthesis pathways. Such findings identify different classes of putative sncRNA biomarkers in serum associated with age and age associated pathologies; these sncRNA may function as epigenetic regulators to modify transcription of target genes involved in key molecular regulatory pathways. Collectively, our findings characterize for the first‐time the global sncRNA landscape in serum from older individuals and provide novel targets for interventions aimed at improving ageing and health trajectories in older age.

The analysis of sncRNA expression profiles identified four major sncRNA subtypes in serum from older individuals, including miRNAs, tRNAs, tRFs, and piRNAs. The expression of two miRNAs were found to be differentially expressed with age, *miR‐375/miR‐375‐3p* and *miR‐769/miR‐769‐5p*. *miR‐375/miR‐375‐3p*, a 22‐nucleotide mature miRNA located on the reverse strand of chromosome 2 was downregulated with age. *miR‐375* was first described in pancreatic islet cells where it functions as an important regulator of β cell development and function.^39^, ^40^ However, more recently *miR‐375* has been shown to function in a diverse number of cellular pathways, with changes in *miR‐375* expression reported in cancer, inflammation, autoimmune, cardiovascular diseases, and diabetes.^41^ For example, Xu et al., showed that *miR‐375‐3p* suppresses tumorigenesis and partially reverses chemoresistance by downregulating Hippo signaling through modulation of the Hippo‐YAP1 pathway downstream genes CTGF, cyclin D1, and BIRC5.^42^ The Hippo signaling pathway has also been linked to development, stem cell regulation as well as ageing pathways.^42^, ^43^, ^44^, ^45^ Furthermore, *miR‐375* has been reported to directly target FOXO1,^46^ which has been shown in both experimental models and humans studies to play an important role in longevity through the regulation of cellular processes such as insulin and insulin‐like growth factor signaling, metabolism, autophagy, DNA damage repair, and oxidative stress resistance,^47^, ^48^ Thus, downregulation of *miR‐375/miR‐375‐3p* may reduce repression of target genes within Hippo/FOXO signaling pathways with impacts on ageing and longevity. The second miRNA strongly associated with age was *miR‐769/miR‐769‐5p* which has been reported to be upregulated in many cancers; here expression of *miR‐769/miR‐769‐5p* was increased with age. A number of studies have also reported changes to circulating miRNA expression with age, with for example miR‐17, miR‐19b, miR‐20a, and miR‐106a identified across a range of ageing models.^49^ The miRNAs associated with age identified in this study have not been reported in other ageing studies to date, although their target genes were enriched in ageing related pathways; here however we have assessed serum miRNAs using a non‐targeted approach and in aged individuals over a relatively narrow age range, which may account for the differences observed. Interestingly, even over the narrow age range of individuals assessed in this study a strong linear relationship was observed between the expression of these miRNAs and chronological age, whether this reflects large differences in the ageing processes between older individuals during this period of the life‐ course will need to be determined using a larger number of individuals across a wider age range.

Pathway analysis showed that lysine degradation and central carbon metabolism were enriched amongst the gene targets of *miR‐769/miR‐769‐5p*, suggesting altered expression of these miRNAs maybe associated with changes in metabolism during ageing. Metabolic alterations have been shown to contribute to aging,^50^, ^51^, ^52^, ^53^ with ageing clocks recently developed based upon some of the characteristic metabolic changes observed with age.^54^ For example, during ageing glucose metabolism shifts from aerobic to anaerobic where oxygen consumption and ATP synthesis are not tightly coupled, leading to a reduction in ATP availability, and increased ROS production. Abnormally high levels of ROS can directly induce genomic instability and increase HIF‐1α levels, promoting metabolic programming towards the Warburg effect.^55^ As the miRNAs identified in this study associated with age regulate mRNAs with key regulatory roles in both longevity and metabolic pathways, the altered expression of miRNAs in the present study could either reflect ageing dependent changes in metabolism and/or be part of the casual pathway by which the changes in metabolism occur.

Along with differential expression of miRNAs, 58 tRNAs and 14 tRFs were also associated with age, demonstrating an age‐associated epigenetic profile that exists across multiple sncRNA subtypes. Of the 14 tRFs differentially associated with age, *tRF‐16‐RPM830D* (*tRF‐5019a, tRF‐5019b*) and *tRF‐31‐87R8WP9N1EWJ0* (*tRF‐5030c*) have previously been characterized within tRFdb. *tRF‐16‐RPM830D* (*tRF‐5019a*, *tRF‐5019b*) a 5′‐tRF, a class of tRF produced from mature tRNAs by cleavage of the 5′ end in the D‐loop,^56^ was upregulated with age and has been associated with a number of gene targets involved in pathways linked with the regulation of small GTPase mediated signal transduction. Small GTPases have been strongly linked to aging pathways, for example the small GTPase Ras is a signaling intermediary of the mammalian insulin/IGF‐1‐signaling (IIS) pathway known to play an evolutionary conserved role in lifespan, through the activation of FOXO transcription factors via inhibition of the lipid kinase PI3K and its downstream target AKT.^57^ Thus, modulation of FOXO signaling pathways by tRFs, together with the miRNAs previously reported could indicate a synergistic effect of different sncRNA populations acting the modulate similar pathways.

Age‐associated changes within the different populations of ncRNAs may suggest crosstalk between sncRNA subtypes to modify cellular communication and cell specific signaling pathways associated with ageing, akin to those observed between long non‐coding RNAs (lncRNAs) and miRNAs of which lncRNAs act as miRNA sponges, modulating its availability to endogenous mRNA targets.^58^, ^59^ Combining available gene targets for the age associated sncRNAs revealed the top pathways enriched amongst the combined age associated sncRNAs included chromatin organization, WNT signaling, cellular response to stress, and oncogene induced senescence, pathways implicated in ageing across a range of cell types,^60^, ^61^, ^62^, ^63^, ^64^ suggesting that the circulating sncRNA signature may reflect the ageing process observed within cells.

Associations were observed between circulating tRNAs, tRFs, and piRNAs and sarcopenia status in older individuals. There were three tRFs significantly associated with sarcopenia, however, only *tRF‐5003c* was currently characterized in the tRF database. Gene targets of *tRF‐5003c* were enriched in pathways involved in neural precursor cell proliferation, this enrichment was driven by the *tRF‐5003c* targets *NOTCH1*, *DISC1*, and *GLI2*, which play key role in cell proliferation and differentiation in a number of different cell types including muscle satellite cells. For example, NOTCH signaling is a key determinant of muscle regenerative potential, with reduced NOTCH activation in satellite cells (SC) associated with a decrease in SC function and impaired muscle regeneration,^65^ while DISC1 is essential for oxidative phosphorylation and^66^ GLI2 is a regulator of both MYF5 and MYOD, key myogenic regulators required for SC differentiation and muscle regeneration.^67^, ^68^ In our study there was a positive association between *tRF‐5003c* and sarcopenia status suggesting that increased *tRF‐5003c* expression may contribute to the reduced expression of *NOTCH, DISC1*, and *GLI2* and impaired muscle function.

The incidence of insulin resistance and type 2 diabetes (T2D) have also been shown to increase with age.^69^ In this study, HOMA2‐IR levels were negatively associated with *miR‐10b* levels. *miR‐10b* has previously been reported to be reduced in muscle from hyperglycemic compared to normoglycemia rats,^70^ and in muscle tissue from twins discordant for T2D.^71^ The top pathways enriched amongst the miRNAs associated with HOMA2‐IR levels were lysine degradation, circadian rhythm, and fatty acid metabolism. Interestingly, a number of human studies have shown that circulating 2‐aminoadipic acid (2‐AAA) levels, a metabolite generated from lysine degradation was associated with obesity, metabolic syndrome, and risk of future T2D.^72^, ^73^, ^74^, ^75^ Furthermore, lysine degradation was one of the pathways enriched amongst the age‐associated sncRNAs, suggesting that both age and HOMA2‐IR may act on the same pathways through sncRNA‐mediated mechanisms, identifying a potentially novel mechanism by which age modulates insulin sensitivity. Alterations in the circadian clock and fatty acid metabolism have also been implicated in mediating the severity of insulin resistance. In patients with T2D, circadian changes in insulin sensitivity were abolished compared to healthy individuals. Furthermore, specific disruption of the muscle clock resulted in diminished insulin sensitivity in the muscle, causing hyperglycemia in the non‐fasting condition and glucose intolerance.^76^ In skeletal muscle, free fatty acids (FFAs) inhibit insulin‐stimulated glucose uptake at the level of glucose transport and/or phosphorylation through mechanisms that involve intramyocellular accumulation of diacylglycerol (DAG) and long‐chain acyl‐CoA, activation of protein kinase C (PKC), and decreased tyrosine phosphorylation of insulin receptor substrate 1/2 (IRS‐1/2). Given the enrichment of fatty acid metabolism amongst the gene targets of the HOMA2‐IR associated sncRNAs, a combined sncRNA and lipidomic signature may have utility as predictive model of IR, as fatty acids are known not only to be important for membrane fluidity but also act as signaling molecules and epigenetic regulators.^77^ There was no overlap between the IR and sarcopenia enriched pathways, although a bi‐directional link between IR and sarcopenia has been suggested,^78^ however the gene targets of the majority of the sarcopenia associated sncRNAs are uncharacterized at present.

The main strengths of this study are that we have characterized, for the first time, the circulating sncRNA landscape of serum from older individuals, identifying potential biomarkers of chronological age, sarcopenia and HOMA2‐IR. Furthermore, changes in expression of sncRNA populations have been linked to regulatory target genes and gene pathways implicated in key biological processes associated with ageing, skeletal muscle regulation, and insulin resistance. However, there remain several limitations to this study. Firstly, although this was an exploratory study using small RNA‐seq to assess the expression of the sncRNAs within serum of older individuals, the sample size was relatively small and this maybe a reason why we only observed nominally significant results in some of the analyses. Secondly, the age range of our participants was limited and confined to older women, due to the availability of samples; further studies using larger numbers of participants over a wider age range, and including both women and men, together with replication in a second independent cohort would provide further insight into the viability of using the sncRNAs identified in this study as biomarkers of ageing across the lifecourse. Although, the target genes of the age associated sncRNAs identified in this study were enriched in processes such as chromatin organization, and senescence which are well established markers of the ageing process observed across many cell types, suggesting circulating sncRNA profiles may reflect tissue ageing and have utility as valuable predictors of chronological and biological age.^79^ Thirdly, due to the high degree of novelty of a proportion of the differentially expressed sncRNAs identified, especially tRFs and piRNAs, which have only recently been identified, it was not possible to identify predicted gene targets, ontologies or pathways and therefore assign specific biological function for all differentially expressed sncRNAs, requiring further investigation to identify the functional role of these sncRNAs. However, the identification of novel sncRNAs within this study associated with age, sarcopenia, and HOMA2‐IR reveals promising biomarkers and provides an opportunity for future biological characterization of predicted gene targets and pathways involved in the regulation of age‐associated muscle dysregulation and insulin resistance.

## 5 CONCLUSION

We identify changes in individual sncRNA populations within human serum that are associated with chronological age, sarcopenia and HOMA2‐IR in older community dwelling women. Furthermore, we identify predicted gene targets of these sncRNAs and pathway enrichment of the predicted genes in important biological processes linked with molecular regulation of each phenotype. These findings support the premise that epigenetic regulatory mechanisms may contribute to ageing, the age‐related decline in glycemic control, and may be important regulators of muscle health in older age. Moreover, they provide a highly novel set of serum‐based biomarkers in humans for the development of intervention strategies which could function to modulate the epigenetic landscape of ageing and associated pathologies.

## AUTHOR CONTRIBUTIONS

Mark A. Burton, Karen A. Lillycrop, and Keith M. Godfrey conceived and designed the research; Mark A. Burton performed the research and acquired the data. Elie Antoun analyzed the data and Mark A. Burton, Elie Antoun, and Karen A. Lillycrop interpreted the data. Mark A. Burton and Karen A. Lillycrop drafted the manuscript. All authors were involved in revising the manuscript.

## FUNDING INFORMATION

This work was supported by grant funding from the Medical Research Council (MC_U47585827, MC_ST_U2055, MC_PC_21003; MC_PC_21001), Arthritis Research UK, Royal Osteoporosis Society, International Osteoporosis Foundation, Cohen Trust, NIHR Southampton Biomedical Research Centre, University of Southampton, and University Hospital Southampton NHS Foundation Trust, NIHR Musculoskeletal Biomedical Research Unit, and University of Oxford. KAL is supported by the Rosetrees Trust, Wessex Medical Trust, Gerald kerkut Trust and Rank Prize K.M.G. is supported by the UK Medical Research Council (MC_UU_20/4), the US National Institute On Aging of the National Institutes of Health (award number U24AG047867), the UK Economic and Social Research Council and the Biotechnology and Biological Sciences Research Council (award number ES/M0099X/), the National Institute for Health Research (as an NIHR Senior Investigator (NF‐SI‐055‐0042)), and through the NIHR Southampton Biomedical Research Centre, and the European Union's Erasmus + Capacity‐Building ImpENSA Project. H.P.P. is supported by the National Institute for Health Research through the NIHR Southampton Biomedical Research Centre. This report is independent research, and the views expressed in this publication are those of the authors and not necessarily those of the NHS, the NIHR, or the Department of Health. The grant funders had no role in the design, collection, analysis, and interpretation of data, writing of the paper, or decision to submit for publication. For the purpose of open access, the author has applied a Creative Commons Attribution (CC BY) license to any Author Accepted Manuscript version arising from this submission.

## DISCLOSURES

K.M. Godfrey and H.P. Patel have received reimbursement for speaking at conferences sponsored by companies selling nutritional products. C. Cooper has received consultancy fees and honoraria from Amgen, Danone, Eli Lilly, GlaxoSmithKline, Medtronic, Merck, Nestlé, Novartis, Pfizer, Roche, Servier, Shire, Takeda, and UCB. NCH reports personal fees, consultancy, lecture fees, and honoraria from Alliance for Better Bone Health, AMGEN, MSD, Eli Lilly, Servier, Shire, Consilient Healthcare, Theramex, and Internis Pharma, outside the submitted work. M.A. Burton, K.M. Godfrey, and K.A. Lillycrop are part of academic research programs that have received research funding from BenevolentAI Bio Ltd., Nestec, and Danone. The other authors declare that they have no conflicts of interest. E.M. Dennison reports personal fees and honoraria (outside the submitted work) from UCB, Pfizer, Lilly and Viatris.

## Supporting information


**Figure S1.**.

**Table S1.**.

## Data Availability

The data that support the findings of this study are openly available on the gene expression omnibus (https://www.ncbi.nlm.nih.gov/geo/), under accession number GSE231785.
