# Supplementary material for: The serum small non‐coding RNA (SncRNA) landscape as a molecular biomarker of age associated muscle dysregulation and insulin resistance in older adults
Source: FASEB J. 2024 Jan 31;38(3):e23423. doi: 10.1096/fj.202301089RR (PMC10952661; doi:10.1096/fj.202301089RR)
Supplement: Supplementary file 1 — Figure S1.. [file FSB2-38-0-s001.docx]

**Supplementary Figures**


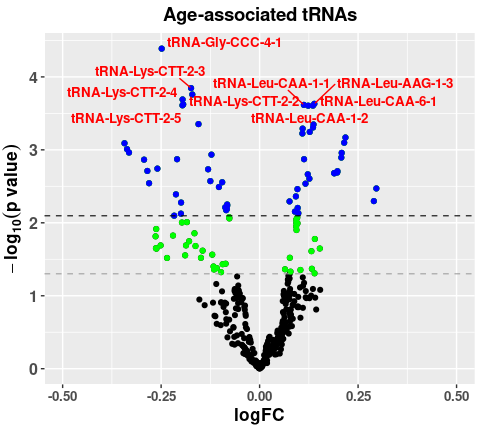

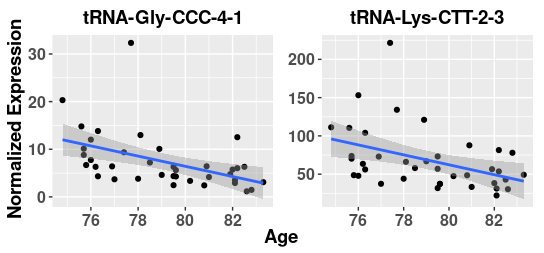

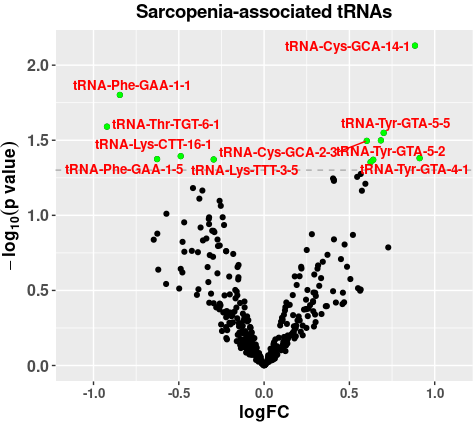

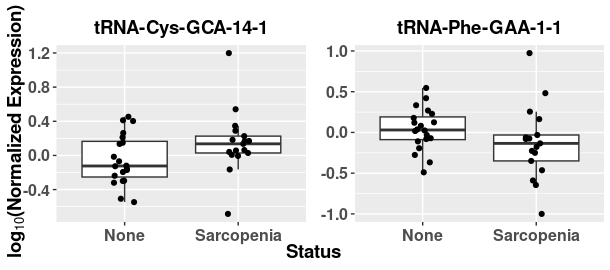

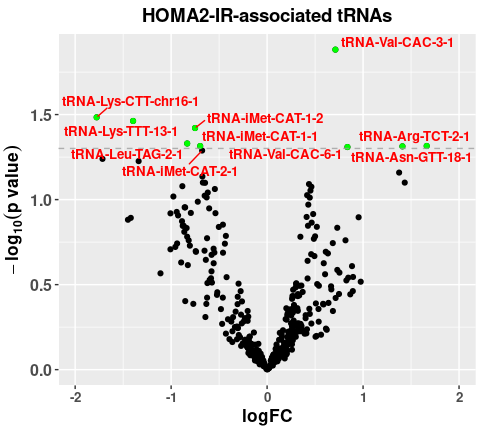

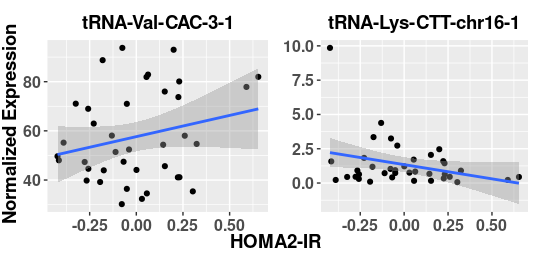


**E**

**D**

**F**

**A**

**C**

**B**

**Supplementary Figure 1:** Volcano plots of Age (**A**), Sarcopenia (**B**) and HOMA2-IR (**C**) associated (P<0.05) tRNAs. Top 2 differentially expressed tRNAs for Age (D), Sarcopenia (E) and HOMA2-IR (**F**) respectively. For volcano plots differentially expressed (P<0.05) sncRNAs are in green and all others in black. Dashed grey line represents p=0.05.
